# Histone β‐hydroxybutyrylation is critical in reversal of sarcopenia

**DOI:** 10.1111/acel.14284

**Published:** 2024-07-30

**Authors:** Qiquan Wang, Xinqiang Lan, Hao Ke, Siman Xu, Chunping Huang, Jiali Wang, Xiang Wang, Tiane Huang, Xia Wu, Mengxin Chen, Yingqi Guo, Lin Zeng, Xiao‐Li Tian, Yang Xiang

**Affiliations:** ^1^ Metabolic Control and Aging Human Aging Research Institute and School of Life Science, Nanchang University, and Jiangxi Key Laboratory of Aging and Diseases Nanchang China; ^2^ Institutional Center for Shared Technologies and Facilities of the Kunming Institute of Zoology, Chinese Academy of Sciences Kunming China; ^3^ Aging and Vascular Diseases Human Aging Research Institute and School of Life Science, Nanchang University, and Jiangxi Key Laboratory of Aging and Diseases Nanchang China

**Keywords:** mitochondria, sarcopenia, skeletal muscle, β‐hydroxybutyrate, β‐hydroxybutyrylation

## Abstract

Sarcopenia, a leading cause for global disability and mortality, is an age‐related muscular disorder, characterized by accelerated muscle mass loss and functional decline. It is known that caloric restriction (CR), ketogenic diet or endurance exercise lessen sarcopenia and elevate circulating β‐hydroxybutyrate (β‐HB) levels. Whether the elevated β‐HB is essential to the reversal of sarcopenia, however, remains unclear. Here we show in both *Caenorhabditis elegans* and mouse models that an increase of β‐HB reverse myofiber atrophy and improves motor functions at advanced ages. β‐HB‐induced histone lysine β‐hydroxybutyrylation (Kbhb) is indispensable for the reversal of sarcopenia. Histone Kbhb enhances transcription of genes associated with mitochondrial pathways, including oxidative phosphorylation, ATP metabolic process and aerobic respiration. This ultimately leads to improve mitochondrial integrity and enhance mitochondrial respiration. The histone Kbhb are validated in mouse model with CR. Thus, we demonstrate that β‐HB induces histone Kbhb, increases mitochondrial function, and reverses sarcopenia.

## Graphical abstract

Sarcopenia is an age‐related muscle disorder characterized by accelerated loss of muscle mass and functional decline, leading to increased disability and mortality globally. This study demonstrates that β‐hydroxybutyrate (β‐HB) effectively reverses myofiber atrophy and improves motor function in Caenorhabditis elegans and mouse models at advanced ages. β‐HB induces histone lysine β‐hydroxybutyrylation (Kbhb), which is crucial for reversing sarcopenia by enhancing mitochondrial pathways, including oxidative phosphorylation, ATP metabolism, and aerobic respiration.

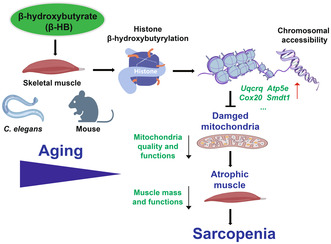

**Abbreviations**
ATP: adenosine 5′‐triphosphate
ChIP‐seq: chromatin immunoprecipitation sequencing
CR: caloric restriction
CSA: cross‐sectional area
DEGs: differentially expressed genes
DMEM: Dulbecco's modified Eagle's medium
GA: gastrocnemius
GEO: Gene Expression Omnibus
GFP: green fluorescent protein
GO: Gene Ontology
GTEx: Genotype‐Tissue Expression
H$E: Hematoxylin and eosin
kbhb: lysine β‐hydroxybutyrylation
KD: ketogenic diet
KEGG: Kyoto Encyclopedia of Genes and Genomes
MyHC: myosin heavy chain
NCBI: National Center for Biotechnology Information
NGM: nematode growth media
OCR: oxygen consumption rate
OE: overexpression
OXPHOS: oxidative phosphorylation
SOL: soleus
TA: tibialis anterior
TNF‐α: tumor necrosis factor‐alpha
β‐HB: β‐hydroxybutyrate

## 1 INTRODUCTION

Sarcopenia is a prevalent age‐related condition that is characterized by a gradual decline in muscle mass, strength, and function. It typically begins to manifest in individuals as early as their fourth decade of life and accelerates in progression after the age of approximately 75 years (Roubenoff & Hughes, 2000). Sarcopenia has a significant impact on the elderly population, contributing to disability, frailty, and a reduced quality of life (Rong et al., 2020, Manrique‐Espinoza et al., 2017). The underlying causes of sarcopenia are complex and involve various factors. Dysregulated signaling pathways, particularly disruptions in mitochondrial homeostasis, which impair the energy production capacity of muscle cells, have been implicated in the onset of this condition (Boengler et al., 2017; Yin et al., 2021). Despite the significant impact of sarcopenia on the aging population, there are currently no approved pharmacological interventions specifically targeting this condition. This highlights the urgent need for alternative approaches to prevent or mitigate the progression of sarcopenia.

Metabolic interventions, including dietary modifications and targeted supplementation of metabolites, have emerged as highly promising strategies for mitigating the onset or progression of age‐related disorders in the last decade (Hamrick & Stranahan, 2020). One of the first identified metabolic interventions that delays sarcopenia is caloric restriction (CR) (Jang et al., 2012). Studies in both aged animal models and humans have supported its effectiveness on preserving skeletal muscle integrity and function (Das et al., 2023; Jang et al., 2012; Lin et al., 2015). In addition to CR, the ketogenic diet (KD), characterized by high fat, low carbohydrate, and moderate protein intake, has garnered attention for its potential health benefits (Han et al., 2020;Puchalska & Crawford, 2017; Xiang et al., 2023). Recent research in aged mice has also shown that the KD can improve skeletal muscle mass and function (Wallace et al., 2021). Both CR and KD induce metabolic shifts that increase ketogenesis and the production of ketone bodies, particularly β‐hydroxybutyrate (β‐HB) (Evans et al., 2017; Lin et al., 2015). Interestingly, exercise, which is a common intervention for sarcopenia, has also been shown to promote ketogenesis and increase β‐HB level (Evans et al., 2017). Despite the increase in circulating β‐HB level through these interventions, the specific role of β‐HB in sarcopenia remains unknown.

In this study, we investigated the effects of β‐HB in sarcopenia using aged *Caenorhabditis elegans* and mice. Our results demonstrated that both β‐HB supplementation and overexpression of its rate‐limiting enzyme have significant protective effects against age‐related sarcopenia. Through RNA‐seq and ChIP‐seq analysis, we discovered that β‐HB‐induced histone β‐hydroxybutyrylation (Kbhb), which enhances the expression of genes associated with mitochondrial pathways and improves mitochondrial function. Importantly, we found that inhibiting histone Kbhb abolishes the protective effects of β‐HB in sarcopenia. These findings provide compelling evidence for the beneficial role of β‐HB and histone Kbhb in reversing sarcopenia, suggesting that targeting β‐HB production and/or Kbhb could be a potential therapeutic strategy for addressing age‐related sarcopenia in humans.

## 2 RESULTS

### 2.1 The capacity for β‐HB production positively associated with muscle mass, but declines with age

**FIGURE 1:**
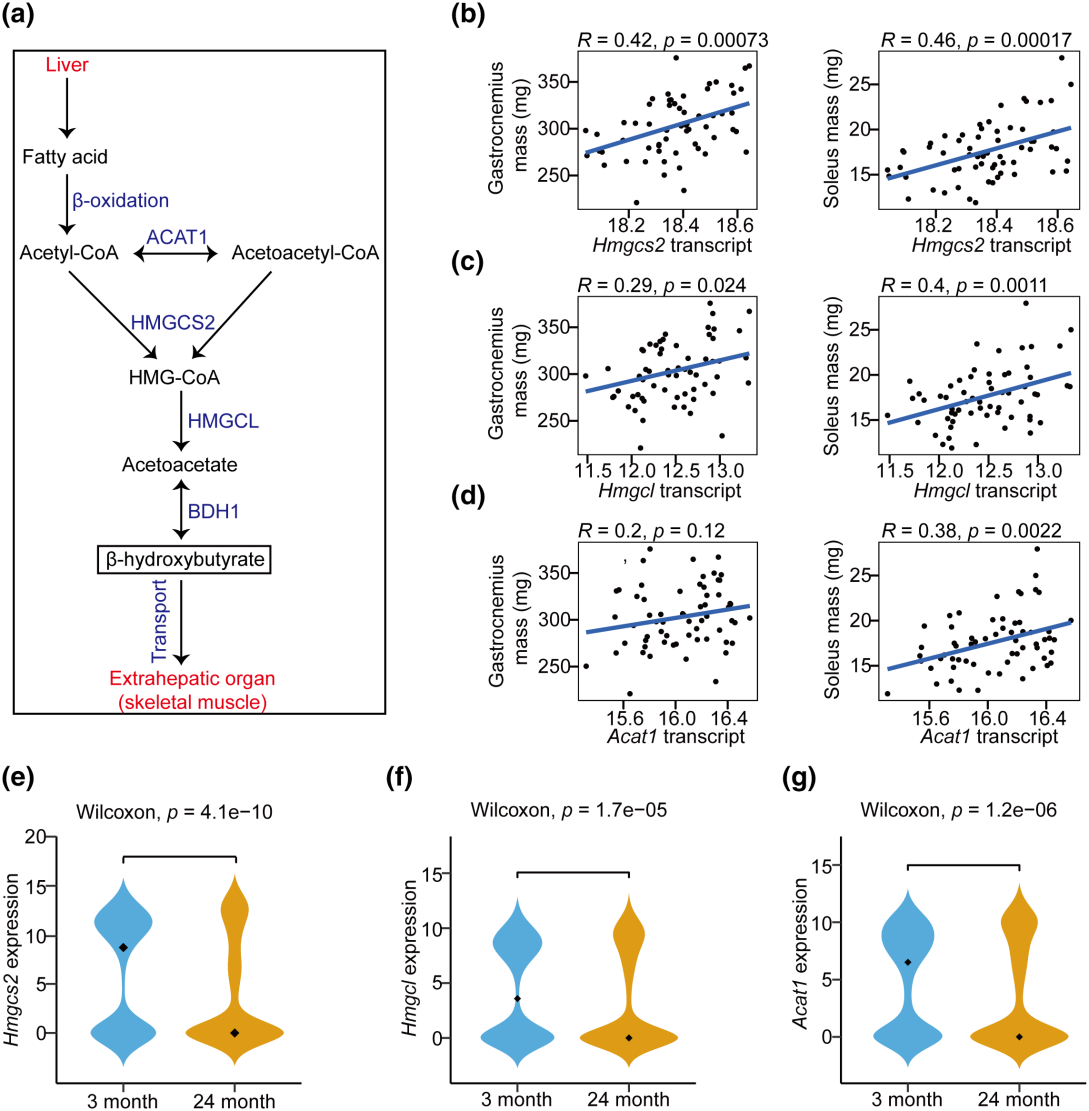
De novo synthesis of β‐hydroxybutyrate (β‐HB) is positively correlated with muscle mass and is inhibited with aging in mice. (a) Schematic of the β‐HB de novo synthesis pathway. (b–d) Pearson correlation of *Hmgcs2* (b), *Hmgcl* (c), and *Acat1* (d) in the liver with measurements of muscle mass in BXD mice (GSE188764). The correlation coefficients (*R*) and significance levels (*p*) are shown. The original *p*‐values are reported, with *p* < 0.0125 (0.05/4, Bonferroni correction) considered significant. (e–g) The single‐cell transcriptome analysis of *Hmgcs2* (e), *Hmgcl* (f), and *Acat1* (g) in the liver of mice at 3 and 24 months of age (GSE109774).

β‐HB is synthesized as an alternative energy source primarily in liver and transported to extrahepatic tissues including skeletal muscle during fasting (Puchalska & Crawford, 2017; Xiang et al., 2023). The expression levels of enzymes, including ACAT1, HMGCS2, HMGCL, and BDH1, serve as indicators of the capacity for β‐HB production (Xiang et al., 2023) (Figure 1a). Among these enzymes, HMGCS2 is the most important key rate‐limiting enzyme for synthesizing the central metabolite HMG‐CoA in β‐HB synthesis (Xiang et al., 2023). We conducted an investigation to examine the correlations between β‐HB production capacity and muscle mass by analyzing liver transcript profiles and muscle mass in the BXD mouse strains, which represent a genetically diverse population (Andreux et al., 2012). Both the mass of gastrocnemius (GA) (*R* = 0.42, *p* = 0.00073) and soleus (SOL) (*R* = 0.46, *p* = 0.00017) were highly positive correlation with the mRNA levels of *Hmgcs2* (Figure 1b). mRNA level of *Hmgcl* and *Acat1* were also positively correlated with the mass of GA and SOL mass (Figure 1c,d), with only GA and *Acat1* expression, as well as the correlation between *Bdh1* expression and both GA and SOL mass, being statistically insignificant (Figure S1).

Since aging is the leading risk factor in the development of sarcopenia (Roubenoff & Hughes, 2000), we next investigated the potential production of β‐HB by examining the change in mRNA levels of enzymes involved in its production using a publicly available dataset (GSE109774) (Schaum et al., 2018). We observed a significant decrease in the transcript levels of *Hmgcs2*, *Hmgcl*, and *Acat1* in aged mice (Figure 1e–g). To validate the diminished potential of β‐HB, we analyzed additional datasets (Aging Atlas) (Aging Atlas Consortium, 2020) and found that this trend was conserved in primates (*Macaca fascicularis*) (Table S1). Moreover, we investigated the β‐HB production potential across different age groups in human liver using the Genotype‐Tissue Expression (GTEx) dataset, and revealed a decreasing trend in the expression of these genes during the aging process in humans, with *ACAT1* showing marginal significance (Figure S2).

The positive correlation between β‐HB production capacity and skeletal muscle mass, along with the decline of this capacity during aging, suggests that either exogenous supplementation or upregulation of its metabolic enzymes may represent an effective strategy for improving sarcopenia.

### 2.2 β‐HB mitigates *in vitro* sarcopenic changes

**FIGURE 2:**
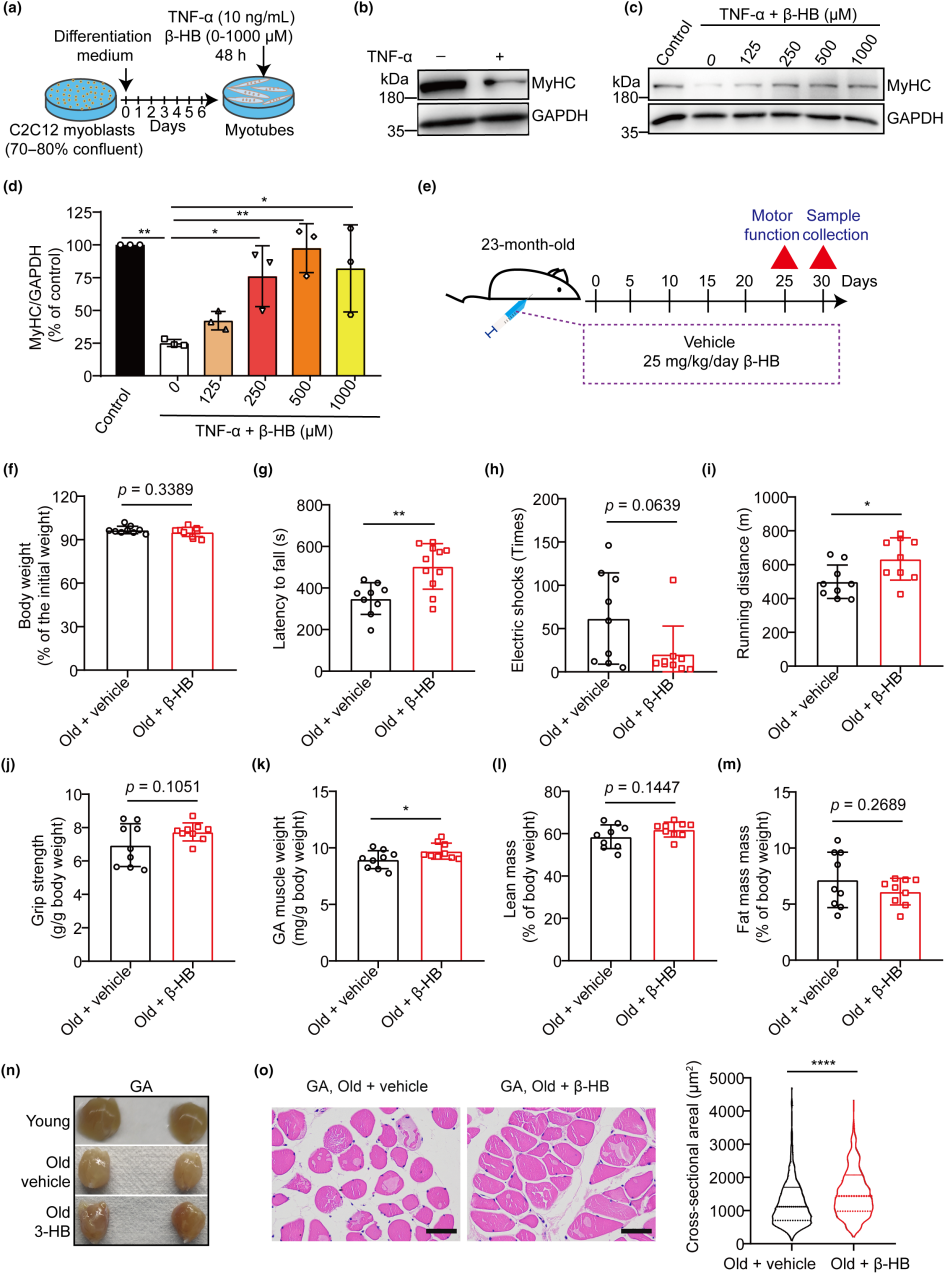
β‐HB protects muscle cells from damage caused by TNF‐α and preserves muscle function and mass in old mice. (a) Schematic diagram for the experimental design of C2C12 myotube damage induced by TNF‐α and intervention with the different concentrations of β‐HB. (b) Western blot analysis of MyHC proteins from C2C12 myotubes treated with TNF‐α. (c) Western blot analysis of MyHC proteins from C2C12 myotubes treated with TNF‐α and different concentrations of β‐HB. (d) Results in (c) were quantified by Image J (*n* = 3). (e) Schematic diagram of the experimental design for treatment of 23‐month‐old mice with β‐HB (25 mg/kg) or vehicle (equal‐volume solvent) by daily intraperitoneal injection for 1 month (*n* = 9). (f) The change of body weight. (g) Latency to fall in the rotarod test. (h) The times of electric shock on the treadmill within 30 min. (i) The running distance was plotted against time to exhaustion. (j) The relative upper limb grip strength. (k) GA muscle weights normalized to body weight. (l) NMR test the body lean percentage of mice. (m) NMR test the body fat percentage of mice. (n) Representative images of the GA muscle. (o) Representative photomicrographs of H&E‐stained GA muscle sections and the distribution of fiber sizes. Scale bar, 50 μm. Values expressed as the mean ± SD. Significance determined using one‐way ANOVA followed by Dunnett's multiple comparison test (d) and unpaired two‐tailed *t* test (f–m and o). *p < 0.05, **p < 0.01, and ****p < 0.0001.

Previous epidemiological studies have consistently shown a strong association between sarcopenia in older adults and elevated levels of proinflammatory cytokines, particularly tumor necrosis factor‐alpha (TNF‐α) (Koliaki et al., 2019; Xu et al., 2022). To mimic the *in vitro* conditions of sarcopenia, we chose the mouse myoblast cell line C2C12 as our research model. Upon differentiation, C2C12 myotubes were treated with or without the TNF‐α (Figure 2a), which is commonly used as an *in vitro* model for studying sarcopenia (Wu et al., 2023). Western blot analysis revealed a significant decrease in myosin heavy chain (MyHC) content under this atrophic condition (Figure 2b), confirming the successful construction of our *in vitro* research model. To determine if β‐HB could protect against C2C12 myotube damage, we treated the cells with various concentrations of β‐HB (0–1 mM) along with TNF‐α (Figure 2a). Western blot analysis showed that β‐HB had a significant dose‐dependent preventive effect on the decrease in MyHC protein level induced by TNF‐α (Figure 2c,d). We also investigated the direct effects of β‐HB at concentrations ranging from 0 to 1 mM on the differentiation and post‐differentiation stages of C2C12 myotubes. The results showed that β‐HB did not increase the MyHC protein levels under either condition (Figure S3). These findings suggest that β‐HB treatment prevented sarcopenic changes *in vitro*.

### 2.3 Declines in muscle function and fiber size were reversed by β‐HB in aged mice

We conducted further investigations into the protective effects of β‐HB on sarcopenia in 23‐month‐old mice. These mice were divided into two groups: one group was administrated with β‐HB (25 mg/kg/day), while the other group received PBS as a vehicle control (Figure 2e). Although there were no significant differences in body weight between the two groups, β‐HB treatment resulted in a slight decrease in body weight in aged mice (Figure 2f). To assess the impact of β‐HB on muscle function, we conducted functional tests including the rotarod test, running test, and grip strength test. Our findings indicate that β‐HB treatment enhanced exercise endurance and motor coordination in aged mice compared to those treated with the vehicle (Figure 2g–i). However, in the grip strength tests, while β‐HB treatment showed a trend towards increased strength, this difference did not reach statistical significance (Figure 2j).

To investigate whether the beneficial effects of β‐HB in aged mice are associated with changes at the individual muscle fiber level, we isolated skeletal muscles from aged mice treated with β‐HB. We found that β‐HB increased the relative weight of the GA muscle (Figure 2k). The results from the body composition analysis using NMR also partially validated these observations, showing a slight increase in lean mass and a slight decrease in fat mass (Figure 2l, m; Figure S4). Interestingly, the muscles from old mice treated with β‐HB exhibited a redder color compared to those from control mice, resembling the color of muscles from 8‐week‐old young mice (Figure 2n). The red color in muscle tissue is attributed to myoglobin, a protein that stores and transports oxygen, indicating well‐oxygenated and active muscles (Garcia et al., 2018; Kim et al., 2020; Song et al., 2022). The effects of β‐HB on the weight and redness of the GA muscle suggest its potential benefits in improving muscle fibers. We further assessed the cross‐sectional area (CSA) of the GA muscle using H&E staining. The results showed an increase in CSA with β‐HB treatment (Figure 2o). These findings demonstrate that supplementation with β‐HB effectively mitigates sarcopenic change in aged mice.

### 2.4 β‐HB enhances motor function and preserves myofiber integrity in aged *C. elegans*

**FIGURE 3:**
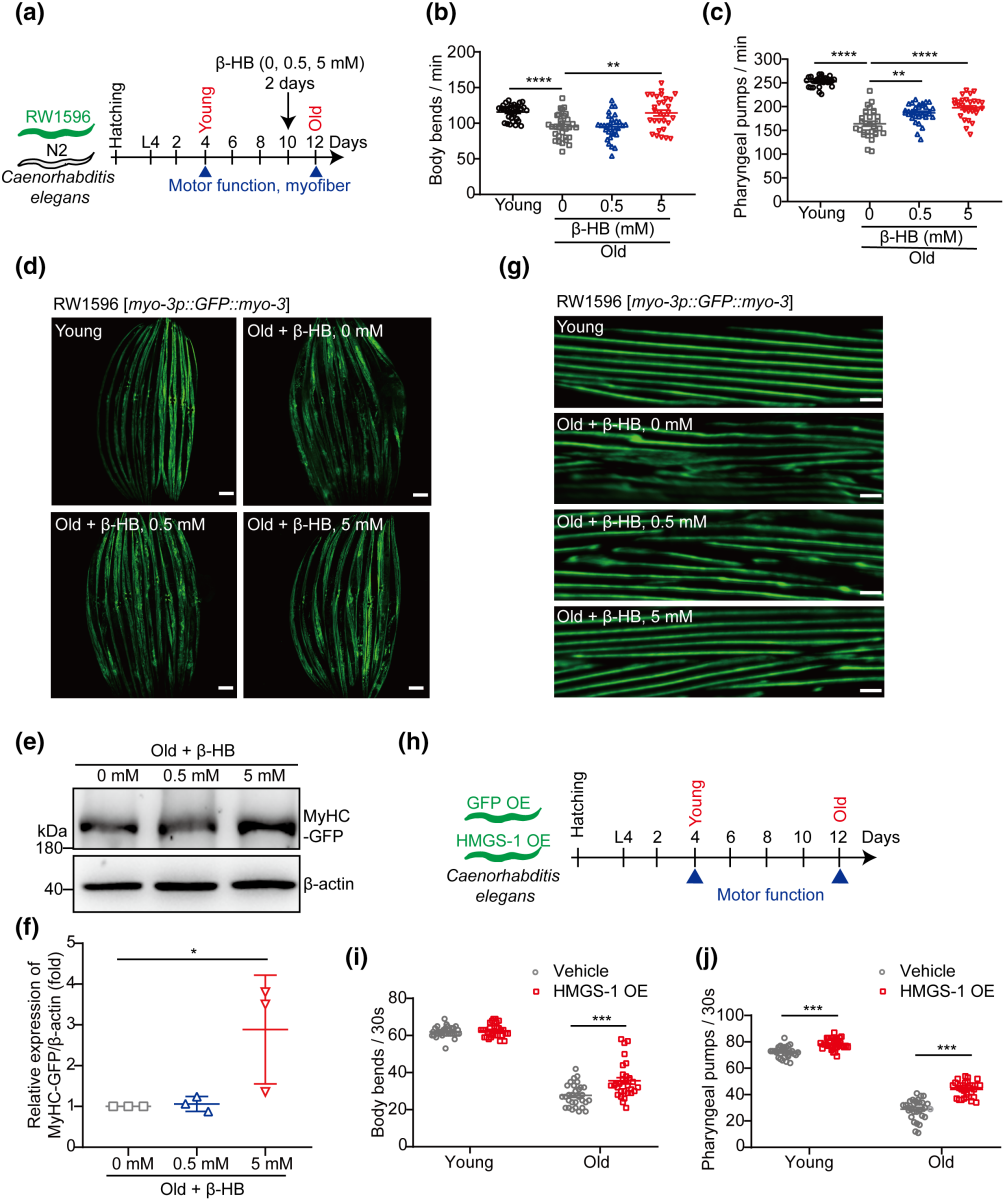
Treatment of β‐HB and overexpression of HMGS‐1 enhance motor functions and alleviate myofilament abnormalities in aged worms. (a) Schematic illustration of the experimental design for β‐HB treatment of wild‐type (N2) and RW1596 nematode strains. (b and c) Motor functions of N2 worms cultured on *E. coli* OP50 lawns on NGM plates supplemented with different concentrations of β‐HB, an evaluated by body bends (b) and pharyngeal pumps (c) (*n* = 30). (d) Representative images of young and old RW1596 worms expressing GFP in body wall muscle under treatment with different concentrations of β‐HB. GFP represents the distribution of myofilaments. Scale bars, 100 μm. (e) Western blot analysis of protein expression of MyHC fused to GFP (MyHC‐GFP) in the β‐HB treatment groups using anti‐GFP antibody. (f) Results in (e) were quantified by Image J (*n* = 3). (g) Representative images of individual fibers from young and old RW1596 worms treated with different concentrations of β‐HB. Scale bars, 5 μm. (h) Schematic illustration of the experimental design for GFP OE (vehicle control) and HMGS‐1 OE worms under normal culture conditions. (i and j) Motor functions of HMGS‐1 OE and vehicle control worms cultured under normal conditions evaluated by counting body bends (i) and pharyngeal pumps (j). (*n* = 30). Values expressed as the mean ± SD. Significance determined using one‐way ANOVA followed by Dunnett's multiple comparison test (b, c, and f) and unpaired two‐tailed *t* test (i and j). *p < 0.05, **p < 0.01, ***p < 0.001, and ****p < 0.0001.

*C. elegans* is a widely used model organism for studying genetics and biomedical research, particularly in the field of aging and age‐related diseases (Zhang et al., 2020). Similar to mammals, sarcopenia is also observed in *C. elegans* as a consequence of age‐related functional decline (Lehmann et al., 2017). Over the past decade, *C. elegans* has emerged as a valuable model for investigating sarcopenia (Christian & Benian, 2020). To investigate the functional conservation of β‐HB in its protective effects against sarcopenia, we cultured aged worms (Day 10) and treated them with different concentrations of β‐HB (0, 0.5, and 5 mM) for 2 days (Figure 3a). We evaluated worm locomotor ability by measuring body bends and pharyngeal pumps. In the wild‐type N2 strain, we observed an age‐related decline in body bends and pharyngeal pumps. However, treatment with β‐HB in a dose‐dependent manner mitigated these signs of declining motor function in aged worms (Figure 3b,c).

In addition to motor function decline, aging in *C. elegans* is also associated with myofilament abnormalities (Lehmann et al., 2017). Therefore, we further assessed the effect of β‐HB supplementation on muscle architecture using a transgenic myo3::GFP reporter strain (RW1596), which expresses GFP‐tagged MyHC A in body wall muscle cells (Figure 3a). Consistent with the results of motor function, supplementation with 5 mM β‐HB significantly increased the total level of myo3 protein in myofibers (Figure 3d–f, Figure S5) and improved myofilament integrity in aged worms (Figure 3g). These findings demonstrate the effectiveness of β‐HB supplementation in delaying age‐related sarcopenia in *C. elegans*.

Taken together, these results indicate that β‐HB treatment exhibits a conserved protective effect, reversing age‐associated muscle atrophy in multiple models.

### 2.5 Increasing endogenous β‐HB production capacity improves motor function in aged *C. elegans*

In mammals, the production of β‐HB is primarily regulated by the activation of the rate‐limiting enzyme HMGCS2 (Puchalska & Crawford, 2017). The aforementioned results have demonstrated a positive correlation between the expression of HMGCS2 and muscle mass in mice (Figure 1b). In *C. elegans*, HMGS‐1 is the ortholog of HMGCS2 in mammals (Figure S6a). To further investigate the endogenous potential beneficial effects of enzymes involved in β‐HB production in sarcopenia, we overexpressed *hmgs‐1* fused to *gfp* in the worms (*pact1*::*hmgs‐1*::*GFP*). qPCR and immunofluorescence analysis confirmed the successful generation of the transgenic HMGS‐1 overexpression (HMGS‐1 OE) strain (Figure S6b,c). Consistent with the results obtained from exogenous β‐HB treatment, the aged HMGS‐1 OE worms exhibited improved motor function compared to the control worms. This was evident from the increased number of body bends and higher pharyngeal pump frequency observed (Figure 3h–j). These findings suggest that enhancing the capacity of β‐HB production, such as activating the enzyme involved in β‐HB production, can also lead to improvements for sarcopenia.

### 2.6 β‐HB enhances transcription of genes associated with mitochondrial pathways and increases mitochondrial function

**FIGURE 4:**
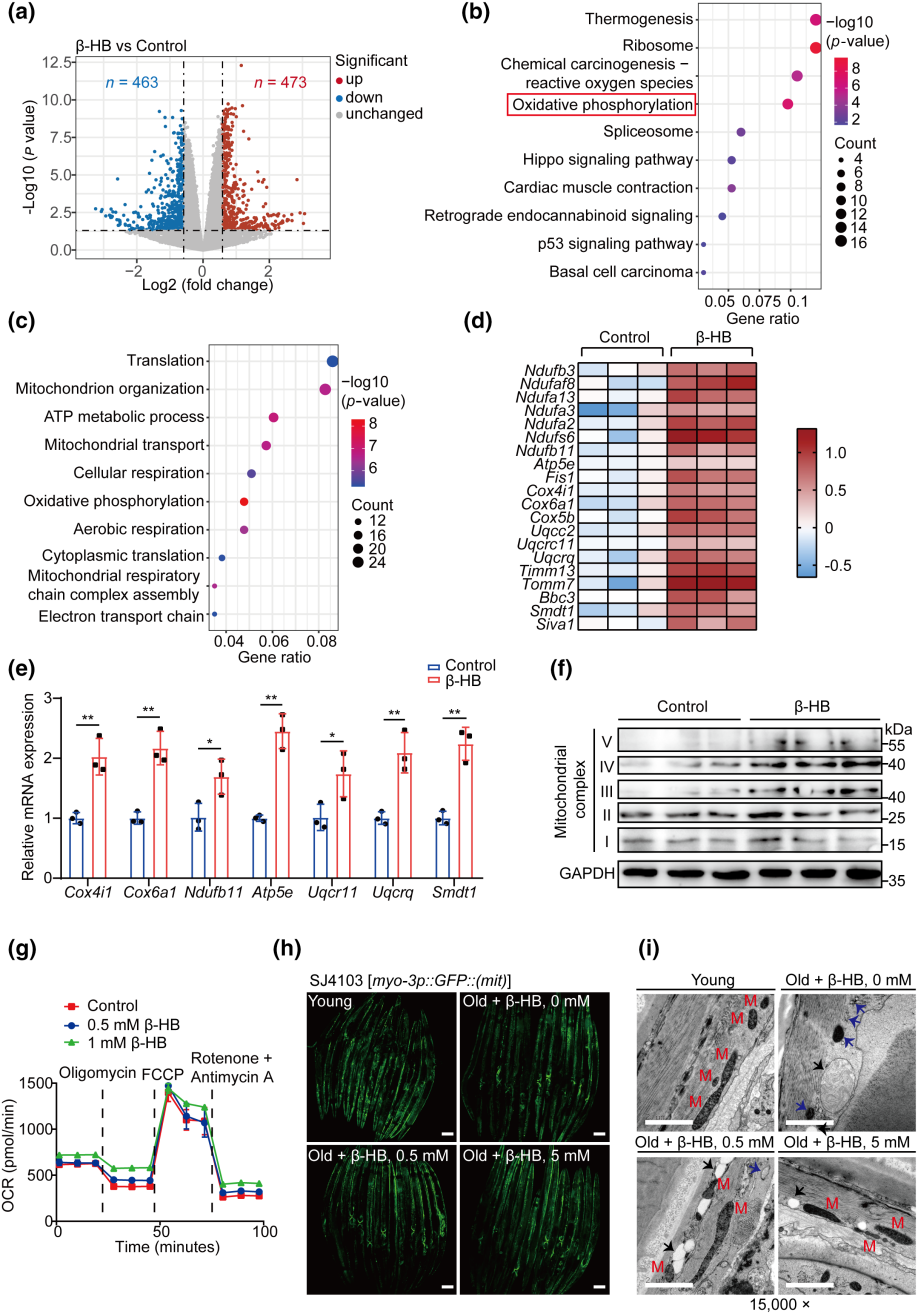
β‐HB enhances transcription of genes associated with mitochondrial pathways and improves mitochondrial function. (a) Volcano plot of DEGs identified in β‐HB and PBS control treated C2C12 myotubes, as determined by RNA‐seq. (*n* = 3). Genes with *p*‐values smaller than 0.05 and fold changes >1.5 were regarded as significantly changed (Red: Upregulated; Blue: Downregulated). (b and c) KEGG (b) and GO enrichment pathways (c) of up‐regulated genes in C2C12 myotubes treated with β‐HB, the top 10 enriched pathways excluding disease‐related pathways are shown. (d) Heatmap of mitochondrial pathway‐related genes identified in (b and c). (e) Expression level of mitochondrial pathways‐related genes in C2C12 myotubes measured by qPCR (*n* = 3). (f) Western blot analyses of the levels of five OXPHOS complexes in C2C12 myotubes treated with PBS or β‐HB. (g) OCRs of C2C12 myotubes treated with different concentrations of β‐HB. (h) Confocal microscopy visualization of mitochondria in body wall muscle cells from GFP‐expressing SJ4103 worms treated with different concentrations of β‐HB. Scale bar, 100 μm. (i) Transmission electron microscopy visualization of mitochondrial morphology in N2 worm body wall muscle treated with different concentrations of β‐HB. Normal mitochondria (M), empty mitochondria (black arrows), and aberrant mitochondria (blue arrows). Scale bar, 2 μm. Values expressed as the mean ± SD. Significance determined using unpaired two‐tailed *t* test (e); *p < 0.05 and **p < 0.01.

To investigate the cellular mechanisms by which β‐HB regulates sarcopenia, we treated C2C12 myotubes with β‐HB and conducted transcriptomic analysis. We identified a total of 936 differentially expressed genes (DEGs), with 473 genes upregulated and 463 genes downregulated (Figure 4a and Table S2). To gain further insights into the functional implications of these gene expression changes, we performed Kyoto Encyclopedia of Genes and Genomes (KEGG) and Gene Ontology (GO) enrichment analysis and revealed that β‐HB positively regulates pathways related to mitochondria, including mitochondrial oxidative phosphorylation (OXPHOS), mitochondrion organization, adenosine 5′‐triphosphate (ATP) metabolic process, and cellular respiration (Figure 4b–d). To validate the findings from the transcriptomic data, we performed qPCR to confirm the expression changes of selected mitochondrial pathway‐related genes, such as *Cox4i1*, *Cox6a1*, *Ndufb11*, and *Atp5e*, in C2C12 myotubes treated with β‐HB. The qPCR results were consistent with the transcriptomic data (Figure 4e). These results were further confirmed by Western blot analysis, which showed an upregulation of the OXPHOS complexes (Figure 4f).

Mitochondrial dysfunction has been implicated in age‐related muscle loss (Boengler et al., 2017). To further explore the effects of β‐HB on mitochondria, we first analyzed the oxygen consumption rate (OCR) of C2C12 myotubes and found treatment of β‐HB increased mitochondrial respiration (Figure 4g). We further investigated the influence of β‐HB on age‐related mitochondrial dysfunction using *pmyo‐3*::*GFP* SJ4103 worms expressing GFP at high levels in the mitochondria of body wall muscle. Compared with the young group, the aged group showed an age‐dependent decrease in mitochondrial content based on the decreased GFP level. However, aged worms exposed to β‐HB showed an improvement in mitochondrial numbers in body wall muscle (Figure 4h). Furthermore, the apparent beneficial effect of β‐HB on mitochondria was reinforced by electron microscopy results. As shown in Figure 4i, the aged worms exhibited a significant number of mitochondria with abnormal morphology. However, β‐HB treatment, especially at 5 mM, attenuated the age‐induced disorganization of mitochondrial morphology and integrity. These findings suggest that β‐HB may enhance the transcription of genes associated with mitochondrial pathways, thereby increasing mitochondrial function and alleviating sarcopenia.

### 2.7 Histone β‐hydroxybutyrylation is central to β‐HB enhancing transcription of genes associated with mitochondrial pathways

**FIGURE 5:**
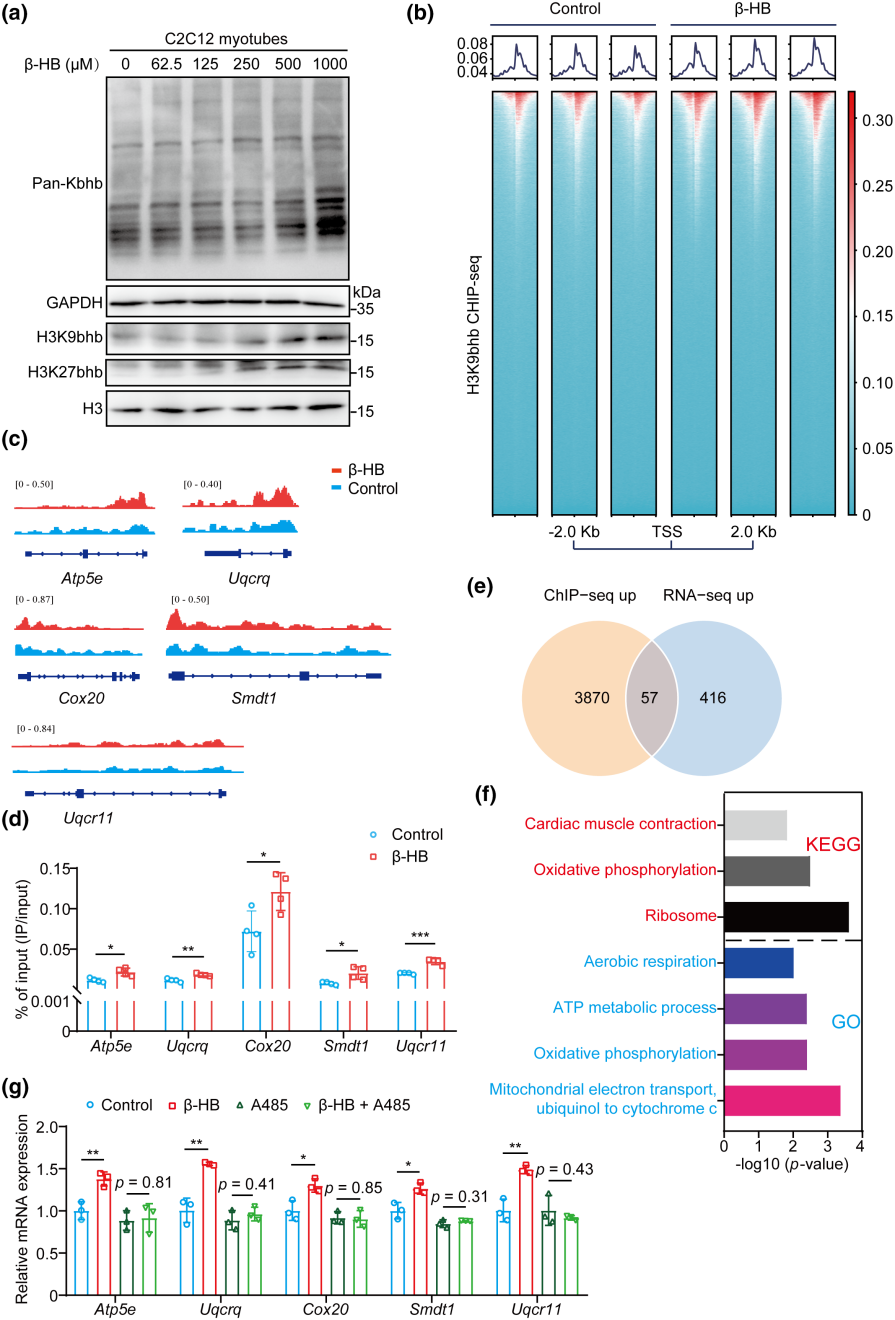
Histone β‐hydroxybutyrylation (Kbhb) is central to β‐HB enhancing transcription of genes associated with mitochondrial pathways. (a) Western blot analysis of pan‐Kbhb, H3K9bhb and H3K27bhb in C2C12 myotubes treated with different concentrations of β‐HB. (b) Heatmaps showing ChIP‐seq density within ±2 kb of the transcription starts site (TSS). Peaks are ranked from high to low H3K9bhb distribution (*n* = 3). (c) ChIP‐seq tracks of H3K9bhb at mitochondrial pathway‐related genes loci. (d) ChIP‐qPCR analysis of mitochondrial pathway‐related genes. (*n* = 4). (e) Venn diagram showing the number of common differentially regulated genes among RNA‐seq and H3K9bhb ChIP‐seq. (f) KEGG and GO enrichment dot plot of 57 common regulated genes among RNA‐seq and H3K9bhb ChIP‐seq. (g) C2C12 myotubes were treated with β‐HB and A485, and the mRNA levels of mitochondrial pathway‐related genes were detected by qPCR. Values expressed as the mean ± SD. Significance determined using unpaired *t* test (d and g); *p < 0.05, **p < 0.01, and ***p < 0.001.

Recent studies have revealed that β‐HB acts as a chromatin regulator, facilitating the modification of histones through lysine Kbhb. This modification is closely associated with active gene transcription, highlighting the crucial role of β‐HB in transcriptional regulation (Terranova et al., 2021; Xie et al., 2016). In this study, Western blot analysis showed an increase in the modification of lysine residues of proteins (pan‐Kbhb), as well as specific histone lysine residues such as H3K9bhb and H3K27bhb, in C2C12 myotubes treated with β‐HB (Figure 5a). To investigate the impact of elevated histone Kbhb marks on gene expression during β‐HB treatment, chromatin immunoprecipitation sequencing (ChIP‐seq) was performed using an antibody against H3K9bhb. The ChIP‐seq results revealed a significant increase in the enrichment of 3927 gene loci, including β‐HB downstream mitochondrial pathway‐related genes such as *Atp5e*, *Uqcrq*, *Cox20*, *Smdt1*, and *Uqcr11* (Figure 5b,c and Table S3). qPCR was performed to validate the findings of H3K9bhb ChIP‐seq, and the qPCR results were consistent with the ChIP‐seq results (Figure 5d). Furthermore, integration of the RNA‐seq and H3K9bhb ChIP‐seq datasets revealed 57 common genes by β‐HB treatment (Figure 5e and Table S4). KEGG and GO enrichment analysis revealed that these 57 common regulated genes were mainly involved in mitochondrial pathways, including oxidative phosphorylation, mitochondrial electron transport, ubiquinol to cytochrome c, ATP metabolic process and aerobic respiration (Figure 5f).

A recent study has identified p300 as the *in vivo* histone Kbhb transferase (Huang et al., 2021). To further confirm that the upregulation of mitochondrial‐pathway related genes in C2C12 myotubes by β‐HB is dependent on histone Kbhb, we treated C2C12 myotubes with A485, an inhibitor of p300 enzyme activity. Despite increasing the expression of *Atp5e*, *Uqcrq*, *Cox20*, *Smdt1*, and *Uqcr11*, the effects of β‐HB were completely abolished by A485 (Figure 5g). These findings strongly suggest that β‐HB enhances transcription of genes associated with mitochondrial pathways in muscle cells by directly inducing Kbhb‐dependent epigenetic modification.

### 2.8 β‐HB‐induced histone Kbhb is indispensable for reversing sarcopenia

**FIGURE 6:**
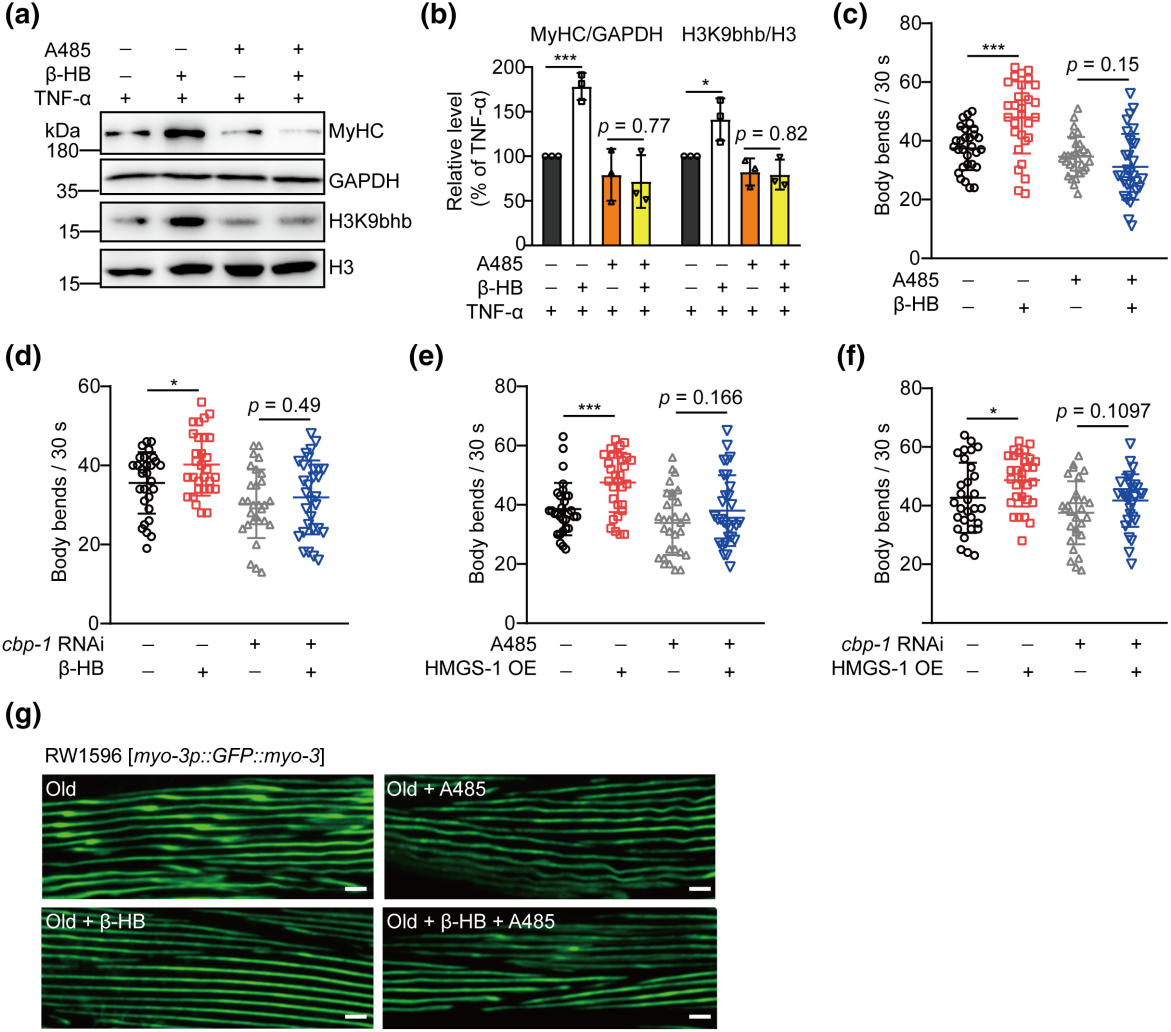
β‐HB‐induced histone β‐hydroxybutyrylation (Kbhb) is indispensable for the reversal of sarcopenia. (a) Western blot analysis of MyHC proteins from C2C12 myotubes treated with TNF‐α, β‐HB and A485. (b) Results in (a) were quantified by Image J (*n* = 3). (c and d) Body bends of aged N2 worms cultured on *E. coli* OP50 lawns on NGM plates supplemented with β‐HB and A485 (c) or *cbp‐1* interference bacteria (d) (*n* = 30). (e and f) Body bends of aged HMGS‐1 OE worms cultured on *E. coli* OP50 lawns on NGM plates supplemented with A485 (e) or *cbp‐1* interference bacteria (f) (*n* = 30). (g) Representative images of individual fibers from young and old RW1596 worms treated with β‐HB and A485. Scale bars, 5 μm. Values expressed as the mean ± SD. Significance determined using unpaired *t* test (b–f); *p < 0.05 and ***p < 0.001.

Mitochondria play a critical role in protecting against sarcopenia (Boengler et al., 2017), and the aforementioned results indicate the significance of histone Kbhb in activating the transcription of genes associated with mitochondrial pathways through β‐HB. To further investigate the potential role of histone Kbhb in mitigating sarcopenia, we first conducted experiments using C2C12 myotubes. The myotubes were treated with A485 or subjected to *p300* RNAi. Subsequently, we assessed the sarcopenic phenotype by the levels of MyHC after treatment with TNF‐α or in combination with β‐HB. The results revealed that β‐HB effectively counteracted the TNF‐α‐induced reduction in MyHC levels. However, either inhibiting or silencing p300 abolished the protective effects of β‐HB treatment on MyHC expression (Figure 6a,b; Figure S7). These findings suggest that histone Kbhb plays a critical role in the β‐HB‐mediated alleviation of sarcopenia *in vitro*.

We next conducted similar experiments using aged worms as a model *in vivo*. We first supplemented aged worms with A485 or introduced *cbp‐1* interference bacteria, which is a homologous functional gene of *p300* in worms. Our results showed that the beneficial effect of β‐HB on improving the motor function ability of aged worms was completely abolished in the presence of A485 or *cbp‐1* interference (Figure 6c,d). Furthermore, the beneficial effect of overexpressing HMGS‐1 on motor function in aged worms was also lost when A485 or *cbp‐1* interference bacteria were introduced (Figure 6e,f). Lastly, we investigated the impact of A485 on the ability of β‐HB to improve muscle fiber structure during aging using RW1596 worms. Consistent with the results of motor function, our findings revealed that the beneficial effect of β‐HB in ameliorating muscle fiber disarray in aged worms was completely abolished upon the addition of A485 (Figure 6g). All these findings suggest that histone Kbhb plays an indispensable role in reversing sarcopenia through β‐HB.

### 2.9 CR increases histone Kbhb in mice skeletal muscle

As an effective intervention for sarcopenia, CR has been reported to increase the levels of β‐HB in mice (Evans et al., 2017). The above results indicate that β‐HB can alleviate the occurrence of sarcopenia by inducing histone Kbhb. To further investigated the relationship between CR, skeletal muscle, and histone Kbhb, we subjected mice to a restricted diet, providing them with only 70% of the amount consumed by ad libitum fed mice, for a period of 2 weeks (Figure S8a). Consistent with the previous findings, our observations revealed that the 2‐week CR diet resulted in a decrease in body weight and fat mass ratio (Figure S8b,c). Additionally, there was an increase in lean mass ratio and relative skeletal muscle mass (Figure S8b–d). Furthermore, we observed elevated levels of β‐HB in CR mice. (Figure S8e). Moreover, Western blot and qPCR analysis demonstrated that CR also led to an increase in the level of H3K9bhb in the skeletal muscle of mice, as well as the expression of downstream genes including *Cox20*, *Uqcr11*, and *Smadt1* (Figure S8f–h). These findings suggest that the beneficial effects of CR on skeletal muscle and sarcopenia may be achieved, at least partially, by raising the level of β‐HB and increasing skeletal muscle histone Kbhb level.

## 3 DISCUSSION

Sarcopenia, the progressive loss of muscle mass and strength that occurs with aging, is still not well understood and lacks an effective pharmaceutical treatment (Pacifico et al., 2022; Roubenoff & Hughes, 2000). Recently, many endogenous metabolites, such as butyrate (Walsh et al., 2015), branched‐chain amino acids (Dos Santos & Anastácio, 2021), and β‐hydroxy‐β‐methylbutyrate (Holeček, 2017) have demonstrated significant health benefits in slowing down the progression of sarcopenia. These findings suggest that endogenous metabolites could be a valuable source for the discovery of new drugs to treat sarcopenia. β‐HB is an important metabolic intermediate that serves as an alternative energy source for extrahepatic tissues when glucose is in short supply (Puchalska & Crawford, 2017; Xiang et al., 2023). In this study, we used *C. elegans* and mouse models to demonstrate that increased levels of β‐HB effectively reduce myofiber atrophy and improve motor function in advanced age. The induction of histone Kbhb by β‐HB promotes the transcription of genes associated with mitochondrial pathways, leading to increased mitochondrial function and the reversal of sarcopenia.

While β‐HB is primarily recognized as an alternative fuel source, recent research has shed light on its role as a crucial signaling metabolite, acting through various pathways. It acts as an endogenous inhibitor of histone deacetylases and can bind to various G protein‐coupled receptors, including HCAR2, and FFAR3 (Puchalska & Crawford, 2017; Xiang et al., 2023). Additionally, β‐HB promotes histone Kbhb, thereby influencing epigenetic processes (Huang et al., 2021; Xie et al., 2016). The multifaceted roles of β‐HB in fuel metabolism and signaling regulation contribute to its involvement in aging, longevity, and age‐related diseases. Several studies have shown that supplementation with β‐HB promote longevity in worms and fruit flies (Edwards et al., 2014; Fan et al., 2021). Moreover, β‐HB treatment has demonstrated protective effects against age‐related diseases such as cancer, neurological disorders, chronic heart failure, and atherosclerosis (Han et al., 2020; Puchalska & Crawford, 2017; Xiang et al., 2023). However, the specific benefits of β‐HB in age‐associated sarcopenia have yet to be fully understood. To the best of our knowledge, our study is the first to report on the potential benefits of β‐HB in alleviating age‐related muscle atrophy. These findings further emphasize the wide‐ranging positive effects of β‐HB in delaying the aging process and intervening in age‐related diseases.

Epigenetic alterations, which play a significant role in regulating gene expression, have been recognized as one of the key regulators of aging and age‐related diseases (López‐Otín et al., 2023). These alterations involve modifications to histones, which are proteins that help orchestrate gene regulation processes (López‐Otín et al., 2023). Numerous studies have investigated the mechanisms linking histone modifications to sarcopenia. For example, acetylation at histone H3K9 and H3K27 decreases with age and is positively correlated with skeletal muscle mass and the expression of MyHC‐IIb (Zhong et al., 2023). In the present study, we identified histone Kbhb as another important epigenetic regulator of sarcopenia. Our data demonstrated that H3K9bhb regulates the expression of genes involved in mitochondrial pathways in skeletal muscle cells. Importantly, we found that disrupting histone Kbhb completely abolished the beneficial effects of β‐HB in sarcopenia. Our findings highlight the significance of epigenetic regulation mediated by histone Kbhb in sarcopenia and provide further insights into the molecular mechanisms of β‐HB involved in this condition. It is noteworthy that β‐HB may exert anti‐muscle atrophy effects through mechanisms beyond improving mitochondrial function. Research indicates that β‐HB benefits protein homeostasis, inflammation, oxidative stress, and skeletal muscle stem cells (Puchalska & Crawford, 2017; Xiang et al., 2023). Further research is needed to determine if these effects contribute to delaying age‐related muscle atrophy.

Based on our analysis of public databases from the BXD mouse strains (Andreux et al., 2012), we have found a positive correlation between the expression of HMGCS2, an enzyme responsible for β‐HB production, and skeletal muscle quality. This suggests that higher levels of HMGCS2 expression may be beneficial for skeletal muscle function. In our study using *C. elegans* as a model organism, we further verified the beneficial roles of HMGS‐1, the ortholog of HMGCS2 in mammals, in alleviating age‐related muscle dysfunction. By overexpressing HMGS‐1 in worms, we observed a significant improvement in the motor functions of aged worms. These results also support previous observations that worms fed with bacteria expressing small interfering RNA against *hmgs‐1* exhibited a significant reduction in pharyngeal pumping levels (Sapir et al., 2014). All of this suggest that mammalian HMGCS2 may serve as a potential target for the treatment of sarcopenia. Therefore, in addition to developing exogenous β‐HB supplements, the development of inducers or activators of HMGCS2 to augment capacity for endogenous β‐HB production may also be an effective approach to improve sarcopenia.

Many lifestyle‐mediated metabolic interventions, such as CR and KD, have been recognized as beneficial for aging and sarcopenia (Das et al., 2023; Puchalska & Crawford, 2017; Xiang et al., 2023). These interventions activate the enzyme HMGCS2, leading to the generation of β‐HB (Evans et al., 2017; Lin et al., 2015). However, the specific role and mechanisms of β‐HB in these interventions remain unclear. In this study, we discovered that CR can generate β‐HB and increase levels of pan‐Kbhb and histone Kbhb in skeletal muscle. Recent studies have also found that KD can increase pan‐Kbhb levels in mouse skeletal muscle (Pathak et al., 2022). Our findings suggest that β‐HB regulates the expression of genes involved in sarcopenia through histone Kbhb in skeletal muscle, thereby delaying the onset of this condition. These insights provide new clues to the mechanisms by which CR and KD can delay the occurrence of sarcopenia and aging. The potential role of histone Kbhb as a mediator of epigenetic modifications in this context is an intriguing area for future research. It is worth noting that, despite the potential benefits of CR and KD for aging and sarcopenia, they also come with corresponding side effects. For example, CR may lead to malnutrition in older individuals (Golbidi et al., 2017), while KD can increase level of plasma cholesterol (Kwiterovich Jr et al., 2003). Taking these factors into consideration, our research may offer a potential alternative approach to CR and KD in counteracting sarcopenia.

It is worth noting that elevated level of β‐HB can be observed not only as a result of lifestyle changes but also in various diseases, including diabetes and acute pancreatitis. While these conditions can potentially lead to ketoacidosis, it typically occurs at higher concentrations of β‐HB (>5 mM) (Wang et al., 2024). Studies have shown that supplementation with exogenous β‐HB or its precursor can significantly improve pancreatic and systemic injuries by inhibiting the activation of proinflammatory macrophages (Zhang et al., 2022). Moreover, there are reports suggesting that β‐HB can inhibit GSK3β and enhance Nrf2 activation in glomerular podocytes, leading to reduced podocyte senescence and injury, improved diabetic glomerulopathy, and decreased albuminuria (Fang et al., 2021). These findings suggest that moderate increases in β‐HB level may have a protective effect in these conditions. Further research is necessary to explore the potential contribution of histone Kbhb modification to this protective effect.

This study has several limitations. First, our reliance on short‐term studies to evaluate β‐HB supplementation limits the assessment of potential long‐term side effects or toxicity. Longitudinal research is crucial to monitor adverse effects on metabolic parameters like lipid metabolism, renal function, and electrolyte balance. Second, findings primarily derived from model organisms require careful interpretation due to physiological and genetic differences from humans. Therefore, validating our findings through rigorous clinical trials or observational studies in human populations is essential to assess the relevance and applicability of β‐HB supplementation in human health. Lastly, the influence of factors such as diet, exercise levels, and individual metabolic variations on the outcomes of β‐HB supplementation has yet to be fully evaluated. Addressing these factors in future research is necessary for a comprehensive understanding of β‐HB's effects in humans.

In summary, our study provides new insights into the roles of β‐HB and histone Kbhb in slowing down muscle atrophy associated with aging. These findings may pave the way for the development of targeted interventions and therapies aimed at mitigating the effects of sarcopenia in humans.

## 4 MATERIALS AND METHODS

### 4.1 Data acquisition and analysis

To analyze the correlation between β‐HB production capacity and muscle mass or aging, the Gene Expression Omnibus (GEO) datasets GSE188764 and GSE109774, representing bulk RNA‐seq and single‐cell RNA‐seq of mouse liver, respectively, were downloaded from the National Center for Biotechnology Information (NCBI) website (https://www.ncbi.nlm.nih.gov/geo/). Pearson correlation was used to assess associations between the expression levels of genes encoding enzymes involved in β‐HB production and muscle mass. Specifically, the genes *Acat1*, *Hmgcs2*, and *Hmgcl* were focused on. The *p*‐values for Pearsons's correlations were calculated based on the *t*‐distribution. To evaluate the relationship between the expression levels of multiple genes and muscle mass, multiple testing correction was applied using the Bonferroni method.

### 4.2 Cell experiments

C2C12 myogenic cells were obtained from Chinese Academy of Sciences cell bank and grown in an incubator at 37°C and 5% CO_2_, and proliferating cells were cultured in Dulbecco's modified Eagle's medium (DMEM) supplemented with 10% FBS (VivaCell). To initiate formation of myotubes, C2C12 myoblasts at 70%–80% confluence was cultured in differentiation medium comprising high‐glucose DMEM supplemented with 2% horse serum (Gibco) for 6 days. To explore the protective effects of β‐HB (Sigma‐Aldrich) against TNF‐α (Novoprotein) induced damage, differentiated C2C12 myotubes were co‐incubated with 10 ng/mL TNF‐α and β‐HB (0, 125, 250, 500, and 1000 μM) for 48 h. To investigate the direct effects of β‐HB on C2C12 myotube differentiation, C2C12 myoblasts at 70%–80% confluence was cultured in differentiation medium with various concentrations of β‐HB (0, 62.5, 125, 250, 500, and 1000 μM) for 6 days. For studying the direct effects of β‐HB on the post‐differentiation stages of C2C12 myotubes, different concentrations of β‐HB (0, 62.5, 125, 250, 500, and 1000 μM) were applied for 48 h. The above treatment process, including differentiation, required daily replacement of the culture medium.

### 4.3 Mouse experiments

C57BL/6 mice were obtained from Changsha Tianqin Biotechnology. The care and experimental protocols for this study were approved by the Institutional Animal Care and Use Committee of Nanchang University (NCULAE‐20221130019). To explore the protective effects of β‐HB on sarcopenia in mice, 8‐month‐old male mice were allowed to naturally age to 23 months and were randomly divided into two groups (9 mice per group): the control group (vehicle‐treated) and the β‐HB (25 mg/kg/day) group. Mice in both groups were intraperitoneally injected daily with either vehicle (PBS) or β‐HB for 1 month. To investigate the effects of CR in mice, 8‐week‐old male mice were randomly divided into two groups (10 mice per group): the control group (ad libitum diet) and the CR group (restricted diet, 70% of the amount consumed by the control group). The body weights of the mice were measured daily. After the behavior tests, the GA, TA, and SOL muscles were rapidly dissected, weighed, and frozen in liquid nitrogen, then stored at −80°C until ready for further analysis, or were fixed in 4% paraformaldehyde and embedded in paraffin.

### 4.4 Treadmill test

The mouse treadmill tests utilized a KW‐PT machine (Karwin, Nanjing, China) equipped with an electrified metal grid positioned at the back of the moving belt. This grid served as a means to deliver an electric shock to the mice if they stopped running. The mice were placed on the treadmill with an initial inclination angle of 10°. The treadmill speed was gradually increased, starting at 8 m/min, then raised to 16 m/min after 10 min, and finally to 24 m/min after 20 min. The treadmill was stopped at the 30‐min mark. The number of electric shocks received by the mice during the 30‐min test period was recorded. The running distance was plotted against time to exhaustion.

### 4.5 Rotarod test

The rotarod machine used for the experiments was a KW‐6C model (Karwin, Nanjing, China), equipped with automatic timers and falling sensors. During the testing phase, the mice were placed on the device at an initial speed of 6 m/min. The speed of the rotating rods was then gradually increased by 2 m/min increments until the mice reached a state of fatigue and fell off the rods. The time and distance at which the mice were able to adhere to the rods before falling were recorded as measures of their motor coordination and endurance.

### 4.6 Grip strength measurement

To evaluate the upper limb grip strength of the mice, a grip strength meter (KW‐ZL‐1, Karwin, Nanjing, China) was utilized. The mice were lifted by their tails and encouraged to grasp onto the firm grids connected to a digital force gauge. The tail of each mouse was gently pulled backwards until the mouse released its grip, and the tension reading on the digital force gauge was recorded as the grip strength. Each mouse underwent five consecutive tests, and the average maximum strength value of the limb muscles (in grams) was calculated.

### 4.7 Body composition analysis

The fat and lean meat rates of mice was analyzed using the Niumag NMR analyzer (QMR23‐060H‐I; Suzhou, China). Put the standard into the instrument for calibration. After weighing the mouse, put it into the detection tube, enter the weight data of the mouse, and analyze the body fat data of the mouse.

### 4.8 Hematoxylin and eosin (H&E) staining

Fixed muscle tissue samples were embedded in paraffin and sectioned into 5‐μm slices. Tissue sections were stained with H&E as described previously (Gao et al., 2019) and visualized by an optical microscope (Zeiss, Germany). The cross‐sectional area of all myofibers was quantified using Image J.

### 4.9 *C. elegans* strains and culture

*C. elegans* strains N2 Bristol (wild type), RW1596 (*myo‐3p*::*GFP*::*myo‐3 + rol‐6*[*su1006*]), SJ410 (*myo‐3*::*GFP*[*mit*]), and *Escherichia coli* OP50, which was used as a food source for worms, were purchased from the Caenorhabditis Genetics Center. The strains that overexpress HMG‐CoA synthase (HMGS‐1) fused to green fluorescent protein (GFP) (*act‐1p*::*hmgs‐1*::*GFP*) and the GFP vehicle (*act‐1p*::*GFP*) were generated at SunyBiotech. All strains were derived from the Bristol N2 strain and were cultured at 20°C on a lawn of *E. coli* strain OP50 plated on 6 cm Petri dishes containing nematode growth media (NGM) agar.

### 4.10 Pharyngeal pumping rate and body movement

For the N2 strain, the nematodes were grown to 10 days of adulthood and transferred to NGM plates containing β‐HB (0, 0.5, and 5 mM) and for 2 days. Next, they were collected to observe the number of pharyngeal contractions during a 60 s interval or were cultured in complete medium to evaluate body movement by observing the bending number for 60 s. For the HMGS‐1 OE strain, the nematodes were grown to 4 and 12 days of adulthood and then transferred to NGM plates. Worms were collected to count the number of pharyngeal contractions in a 30 s interval, or cultured in complete medium to evaluate body movement by counting the number of body bends in a 30 s interval.

### 4.11 Fluorescence measurements of myofibers and mitochondria

RW1596 and SJ4103 worms, which express GFP, were treated with β‐HB (0, 0.5, and 5 mM) at 10‐day‐old adulthood for 2 days. All of the worms were picked in halocarbon oil and then dropped onto a slide coated with a lawn of 2% agarose (thickness, ~1 mm) for visualization of fluorescence under a confocal laser scanning microscope (LSM800, Zeiss, Germany).

### 4.12 Mitochondrial respiration measurements

C2C12 cells were seeded onto sterile XFp24 and 24‐well plates at a density of 3 × 10^4^ cells per well and allowed to differentiate into myotubes for 6 days. After treating the myotubes with β‐HB (0, 500 and 1000 μM) for 48 h, they were sequentially stimulated with 1 μM oligomycin, 0.5 mM carbonyl cyanide 4‐(trifluoromethoxy) phenylhydrazone, and 0.5 μM rotenone. Respiratory parameters, including basal OCR, maximal OCR, OCR for ATP production, total respiratory capacity, spare respiratory capacity, and nonmitochondrial respiration, were calculated. The mito‐stress test generated a report automatically, calculating the XF cell mito‐stress test parameters from the exported Wave data in Excel format.

### 4.13 Transmission electron microscopy

The transmission electron microscopy assay was performed as previously described (Wei et al., 2022). Briefly, N2 worms were treated with β‐HB (0, 0.5, and 5 mM) at 10‐day‐old adulthood for 2 days, then the samples were fixed overnight with 0.1 M PB (pH 7.4) containing 3% paraformaldehyde and 0.1% glutaraldehyde at 4°C, washed with 0.1 M PB four times for 15 min each, and then washed with 0.1 M glycine in 0.1 M PB for 30 min at 4°C. After ethanol gradient dehydration, the samples were embedded in LR white resin (Sigma) and polymerized at 55°C for 24 h. Ultrathin (100‐nm) sections were prepared using an EM UC7 ultramicrotome (Leica Microsystems) and loaded onto 200‐mesh Ni grids (EMCN). The sections were imaged using a JEM1400PLUS transmission electron microscope (Tokyo, Japan).

### 4.14 Western blot

Protein samples were lysed in RIPA buffer containing protease inhibitor cocktail (Yeasen). Total protein lysate concentrations were quantified using a bicinchoninic acid (BCA) assay (BioRAD) according to the manufacturer's protocol. Equal amounts of protein samples were subjected to sodium dodecyl sulfate–polyacrylamide gel electrophoresis and transferred to a polyvinylidene fluoride membrane. The membrane was subsequently blocked with 5% bovine serum albumin dissolved in PBS containing 0.1% Tween 20 (PBST) for 2 h at room temperature, then incubated with the appropriate primary antibody at 4°C overnight, followed by incubation with secondary antibodies for 1 h at room temperature. Protein bands were visualized with Super Signal chemiluminescence reagents (Pierce), as described previously (Wang et al., 2020; Xiang et al., 2014). The protein bands were semi‐quantified using ImageJ software. For data presented as bar graphs, control samples were set as 100%, and the percentage change in intensity is reported.

The primary antibodies were as follows: anti‐MyHC (MF20) antibody (DSHB, diluted 1:100), anti‐GAPDH and anti‐β‐actin antibody (Proteintech, diluted 1:2000), anti‐GFP antibody (Andes, diluted 1:1000), anti–histone H3, anti‐H3K9bhb, anti‐H3K27bhb, pan anti‐Kbhb antibodies (PTM Bio, diluted 1:1000), anti‐p300 antibody (Santa Cruz, diluted 1:1000), anti‐OxPhos antibody cocktail (ThermoFisher, diluted 1:1000). Secondary antibodies were horseradish peroxidase (HRP)‐conjugated goat anti‐mouse secondary antibody and goat anti‐rabbit secondary antibody were purchased from Servicebio (diluted 1:5000), HRP‐labeled goat anti alpaca antibody (Andes, diluted 1:5000).

### 4.15 A485 treatment

For C2C12 experiments, the cells were cultured and differentiated as described above. Subsequently, the differentiated myotubes were co‐incubated with 10 ng/mL TNF‐α, 500 μM β‐HB, and 100 nM A485 (MedChemExpress) for 48 h. The cell sample were harvested for Western blot analysis. For worm experiments, the N2 strain was cultured until 10 days of adulthood and then transferred to NGM plates containing 5 mM β‐HB and 10 μM A485 for 2 days. The HMGS‐1 OE strain was also cultured until 10 days of adulthood and transferred to NGM plates containing 10 μM A485 for 2 days. The worms were then collected to culture in complete medium to evaluate body movement by observing the bending number for 30 s. The RW1596 strain was cultured until 10 days of adulthood and transferred to NGM plates containing 5 mM β‐HB and 10 μM A485 for 2 days. Afterward, the worms were picked for fluorescence visualization using a confocal laser scanning microscope, following the same procedure as described above.

### 4.16 Small interfering RNA synthesis and cell transfection

Small interfering RNAs (siRNAs) targeting *p300* were synthesized by GENERAL BIOL. The siRNA sequences were as follows: si‐*p300*‐1 (5′‐GGAGAGAGGCCCUGAGUUATT‐3′, 3′‐UAACUCAGGGCCUCUCUCCTT‐5′); si‐*p300*‐2 (5′‐AGGAAGAGGAAGAGAGGAATT‐3′, 5′‐UUCUUCUGUUGCUGCAUUGTT‐3′); si‐*p300*‐3 (5′‐GCAUUUGGAUCCAGGAAUATT‐3′, 5′‐UAUUCCUGGAUCCAAAUGCTT‐3′). For cell transfection, we transfected C2C12 myotubes with 150 μM siRNA oligonucleotides using 7.5 μL Lipofectamine 3000 (Invitrogen, Carlsbad, CA, USA) in each well of a six‐well plate. The cell sample were harvested for western blot analysis.

### 4.17 β‐HB measurement

The concentration of β‐HB in mice serum was detected using β Ketone Test Strips following the instructions provided by the manufacturer (FreeStyle).

### 4.18 RNA isolation and qPCR

C2C12 myotubes treated with 500 μM β‐HB for 48 h, and GA muscle samples from CR mice, were immersed in TRIzol reagent and ground using a tissue lyser (Servicebio). RNA extraction was then performed according to the instruction manual. RNA concentration and purity were determined by measurement of optical density at 260 nm using a spectrophotometer (NanoDrop® ND‐ 2000). RNA (1 μg) was reverse transcribed to obtain cDNA using a PrimeScript™ RT reagent kit with a genomic DNA (gDNA) Eraser kit (TaKaRa) according to the manufacturer's instructions. qPCR was performed in a reaction mixture containing cDNA, SYBR Green PCR Master Mix, ddH_2_O and primers, using Real‐time qPCR Detection System (qTOWER3G, AJ, Germany). Data were analyzed by the 2^˗ΔΔCt^ cycle threshold method. Sequences of primers used in the qPCR experiments are shown in Table S5.

### 4.19 RNA‐sequencing

To perform RNA‐seq analysis, we utilized 1 μg of total RNA and followed the TrueLib mRNA Library Prep Kit for Illumina (ExCell Bio) protocol. The RNA was initially separated to isolate the mRNA, which was then fragmented and reverse transcribed into cDNA. Following end repair, the cDNA was barcoded with multiplex adapters, and a cDNA library was constructed. The resulting cDNA libraries were purified using AmpureXP beads, and the samples were quantified using Qubit (Invitrogen) before being sequenced on an Illumina HiSeq platform.

### 4.20 RNA‐seq analysis

For the processing of raw RNA‐seq data, RNA‐seq data obtained from the FASTQ format files were mapped to the mouse reference genome (mm 10), available at (http://genome.ucsc.edu/). Similarly, RNA‐seq data extracted from FASTQ format files of nematodes were mapped to the genome of *C. elegans* (WS245), and reads were counted and summarized using HTSeq. Count data was imported into R (version 4.2.0) and filtered genes. All subsequent statistical and analytical procedures were performed in R (version 4.2.0) and R‐studio (version 2023.09.0).

Initially, evaluated the replicability between samples of different treatments through principal component analysis (PCA) conducted using the prcomp function in R (stats package). Differential gene analysis was performed by estimating the mean and variance of gene expression levels in different groups using the R/Bioconductor package limma (version 3.48.3) via linear modeling. Only transcripts with Fold‐Change ≥1.5 and a *p* < 0.05 were considered significantly DEGs in each group.

### 4.21 ChIP‐seq and ChIP qPCR

ChIP analysis was performed using a SimpleChIP® Enzymatic Chromatin IP Kit (Cell Signaling Technology) according to the manufacturer's instructions. Chromatin was crosslinked with 1% formaldehyde in cell medium at room temperature for 10 min. Glycine was then added to the medium to terminate the reaction. After washing in ice‐cold PBS, 5 × 10^6^ C2C12 myotubes treated with 500 μM β‐HB for 48 h were lysed and sonicated into 150–900‐bp fragments. Immunoprecipitation was performed overnight at 4°C with anti‐histone H3K9bhb and anti‐histone H3. Normal rabbit IgG was used as a negative control. The immunocomplexes were washed using ChIP‐Grade Protein G Magnetic Beads (Cell Signaling Technology) and incubated at 65°C overnight following the addition of 5 M NaCl and Proteinase K to reverse the crosslinks. Free precipitated DNA was further purified. The ChIP and input DNA fragments underwent end‐repair and A‐tailing, followed by adaptor ligation. Subsequently, the DNA libraries were amplified for 15 cycles and subjected to sequencing on the Illumina NovaSeq 6000 platform, using paired‐end 150 as the sequencing mode. The primers used in ChIP‐qPCR analysis were showed in Table S5.

### 4.22 ChIP‐seq analysis

Quality‐filtered ChIP‐seq sequencing reads were aligned to the mouse reference genome (mm 10) and converted to the SAM file format. The SAM files were further transformed into the BAM file format using SAMtools (version 1.3.1). During the analysis of differential peaks, biological replicate BAM files were merged into a single ChIP BAM file or input BAM file. Differential peaks were determined using the R package DiffBind (version 3.8.4) with a significance threshold of Fold‐Change ≥2 and a *p* < 0.05. The nearest genes to the differential peaks were annotated using the ChIP seeker R package (version 1.5.1).

### 4.23 Statistical analysis

The statistical analyses for each figure are detailed in their respective figure legends. For the expression profiling data in Figure 1, the correlation analyses in panels 1b–1d were conducted using Pearson correlation analysis, and *p*‐values were obtained through *t* tests, with multiple testing correction applied using the conservative Bonferroni method. We adjusted the significance threshold to 0.05/N, where N is the number of tests. For Figure 4a, the *p*‐values for each gene were calculated using the limma R package with *t* tests. For Figures 4b,c and 5f, *p*‐values for enrichment analysis were calculated using the hypergeometric test, which evaluates the over‐representation of specific gene sets within the selected list compared to a background set.

For other experiments, all experimental values are expressed as the mean ± standard deviation (SD). Data analysis was performed using Prism v8.0 software. Two‐sample comparisons were conducted using Student's *t* test. Multiple comparisons were performed using one‐way analysis of variance, with post hoc contrasts by Dunnett's multiple‐comparison test. A *p* < 0.05 was considered statistically significant.

## AUTHOR CONTRIBUTIONS

Q.Q.W., X.Q.L., and Y.X. designed the study. Q.Q.W., X.Q.L., S.M.X., C.P.H., J.L.W., X.W., T.E.H., Y.Q.G., and L.Z. performed the experiments. Q.Q.W., H.K., X.W., and M.X.C. analyzed the data. Q.Q.W. wrote the manuscript. X.‐L.T., Q.Q.W., and Y.X. revised the manuscript. All authors read and approved the final manuscript.

## FUNDING INFORMATION

This work was supported by the National Key R&D Program, Ministry of Science and Technology of China (2023YFC3603300, 2023YFF1001000, 2020YFC2002900), the National Natural Science Foundation of China (81873814, 82330046, 32360164), Jiangxi Province (20232ACB215001, 2024SSY07161 and 20221ZDG020070) and Nanchang University (28740105).

## CONFLICT OF INTEREST STATEMENT

The authors declare that there are no conflicts to declare.

## Supporting information


Data S1.

## Data Availability

All data supporting the findings of this study are available in the main text or in the supplementary materials. All sequencing data generated for this study is available at National Genomics Data Center with the following accession numbers: transcriptomics from β‐HB and PBS treated C2C12 myotubes: PRJCA019944; ChIP‐seq of C2C12 myotubes treated with β‐HB and PBS: PRJCA021284.
