# Supplementary material for: Histone β‐hydroxybutyrylation is critical in reversal of sarcopenia
Source: Aging Cell. 2024 Jul 30;23(11):e14284. doi: 10.1111/acel.14284 (PMC11561670; doi:10.1111/acel.14284)
Supplement: Supplementary file 1 — Data S1. [file ACEL-23-e14284-s001.zip › Supplementary Figures and Table 1.docx]

Supplementary Materials for

**Histone β‑hydroxybutyrylation is critical in reversal of sarcopenia**

Qiquan Wang *et al*.

*Corresponding author. Email: Yang Xiang, xiangyang@ncu.edu.cn

Xiaoli Tian, tianxiaoli@ncu.edu.cn

**This file includes:**

Figs. S1 to S8

Table S1

**Other Supplementary Materials for this manuscript include the following:**

Excel files S2 to S5


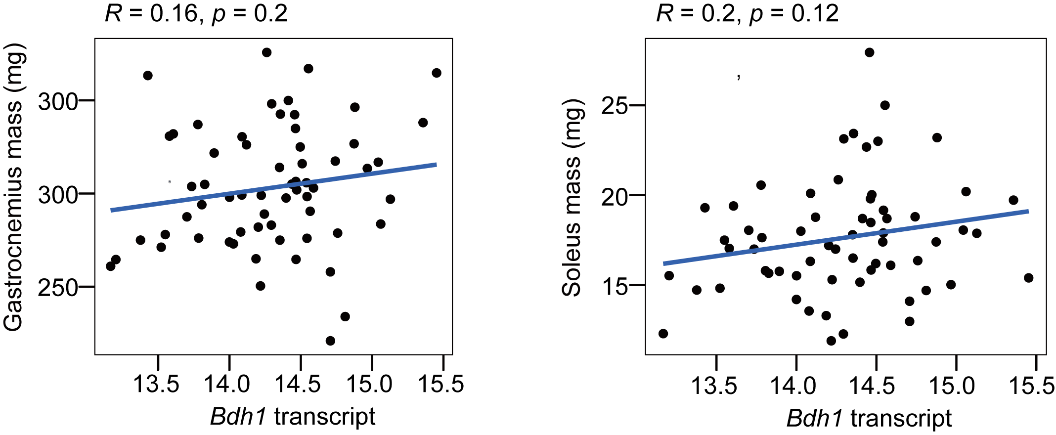


**Supplementary Figure 1.** **Pearson correlation of *Bdh1* in the liver with measurements of muscle mass in BXD mice (GSE188764).**


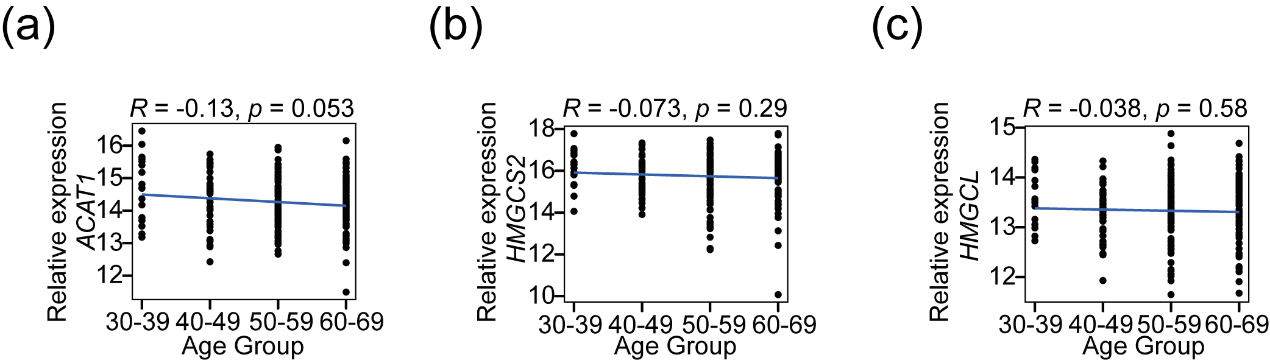


**Supplementary Figure 2.** **De novo synthesis of β-HB exhibits a decreasing trend with aging in human liver. (a-c)** The expression of transcripts of *ACAT1* (a), *HMGCS2* (b) and *HMGCL* (c) among different age groups in human liver (GTEx).


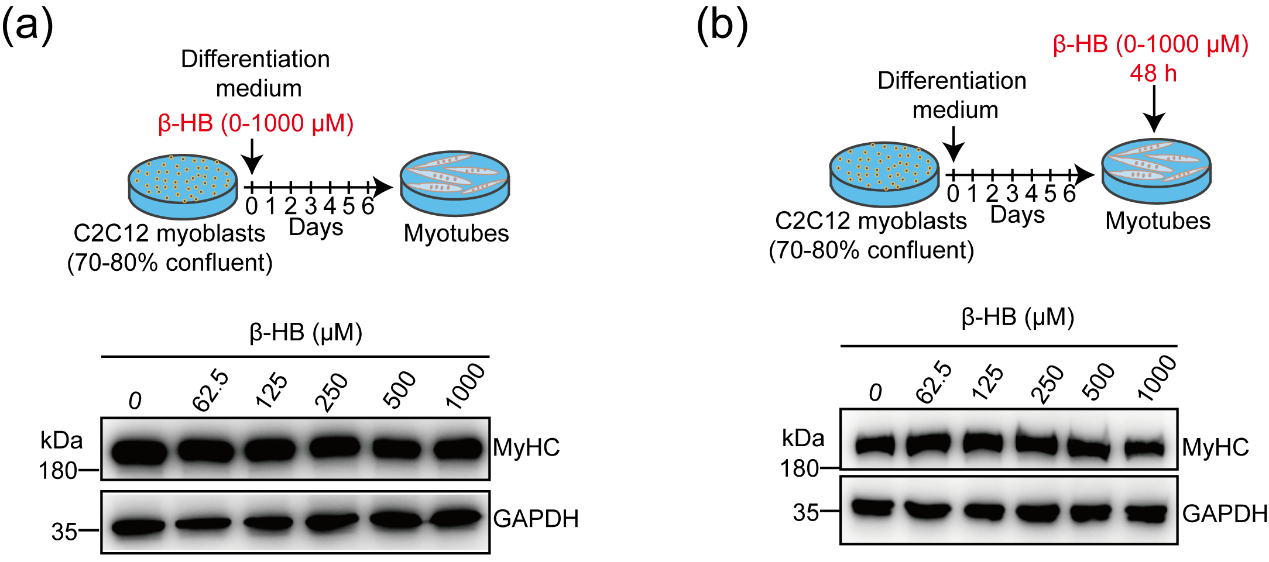


**Supplementary Figure 3. Effects of** **β-HB on C2C12 myotubes differentiation.** **(a)** C2C12 myoblasts were cultured to 70–80% confluent, then were treated with differentiation medium and β-HB (0–1000 μM) together every day for 6 days. The protein levels of MyHC were assessed through western blot analysis. **(b)** The differentiated C2C12 myotubes were treated with β-HB (0–1000 μM) for 48 h. The protein levels of MyHC were assessed through western blot analysis.


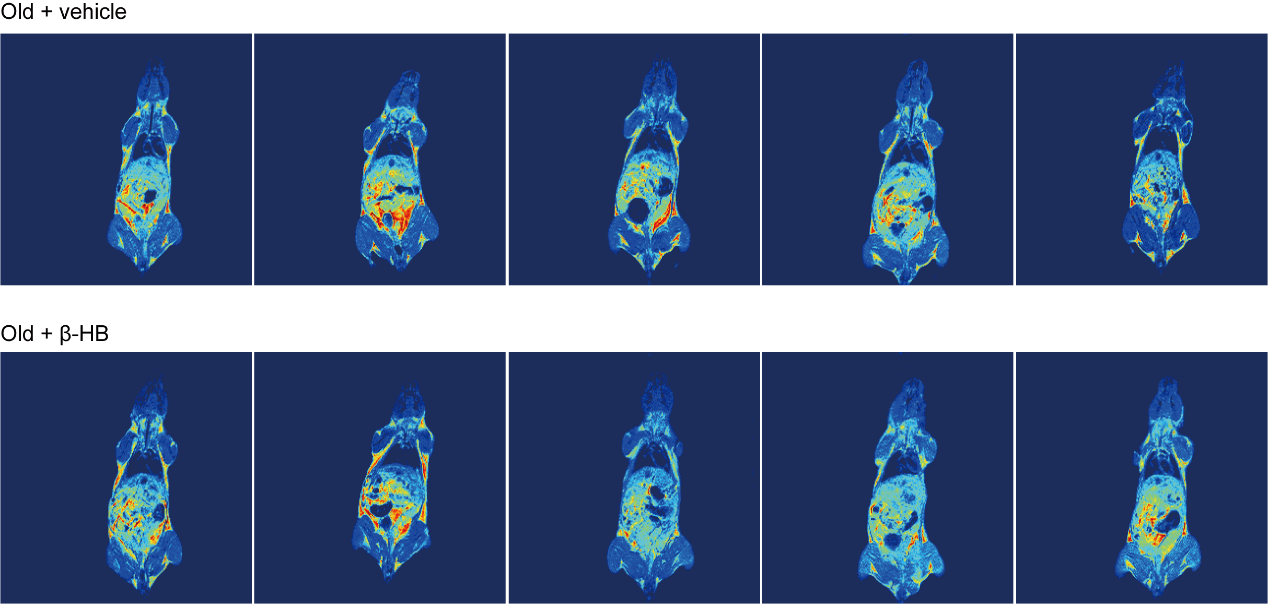


**Supplementary Figure 4. Representative images of control and β-HB-treated old mice analyzed by NMR testing.**


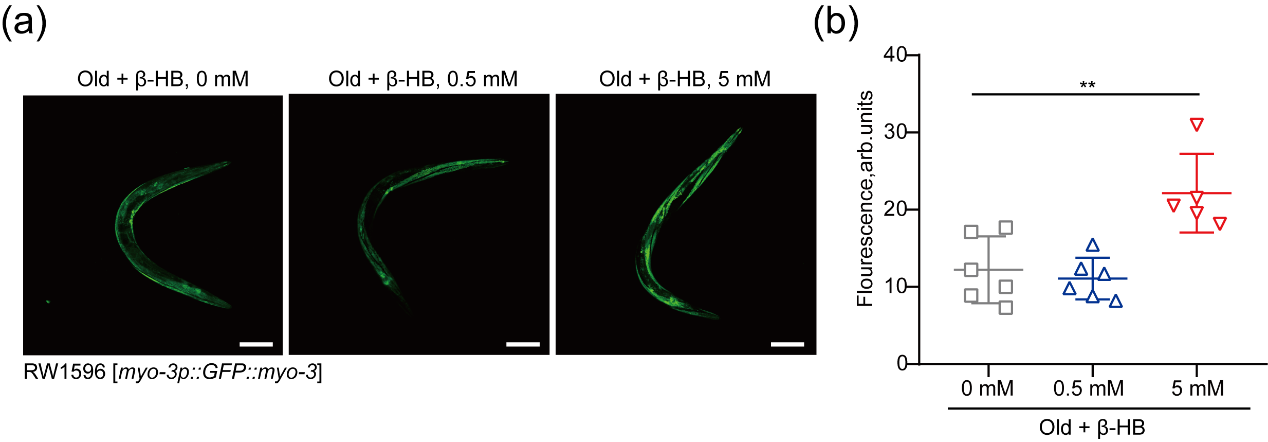


**Supplementary Figure 5. Effects of β-HB on MyHC protein expression in aged worms. (a and b)** RW1596 strain worms expressing GFP in body wall muscle were treated with different concentrations of β-HB at day 10 for 2 days. Representative images of whole worms in each group visualized under confocal microscopy (a) and quantified using ImageJ (b). Scale bar, 100 μm. (n = 5–6). Values expressed as the mean ± SD. Significance determined using one-way ANOVA5–6). Values expressed as the mean ± SD. Significance determined using one-way ANOVA followed by Dunnett's multiple comparison test (b); ***p* < 0.01.


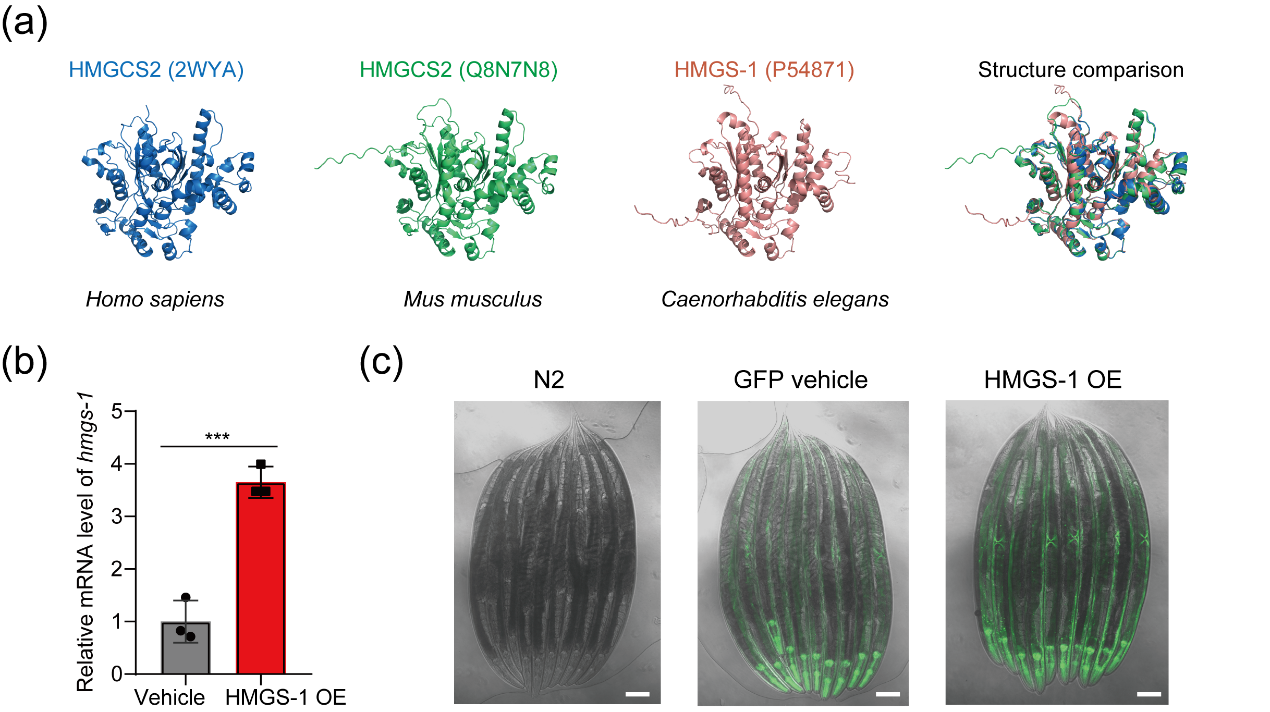


**Supplementary Figure 6. Characterization of HMGS-1 OE worms.** **(a)** Structural comparison of human HMGCS2 (PDB 2WYA), mouse HMGCS2 (UniProt Q8N7N8 by AlphaFold), and *C. elegans* HMGS-1 (UniProt A8X9B5 by AlphaFold) proteins using PyMOL. **(b)** qPCR showing relative mRNA levels of *hmgs-1* in GFP OE vehicle control and HMGS-1 OE worms (n = 3). **(c)** Representative fluorescence micrographs of the wild-type N2, GFP OE vehicle control, and HMGS-1 OE worms. Scale bar, 100 μm. Values expressed as the mean ± SD. Significance determined using unpaired *t* test (b); ****p* < 0.001.

**
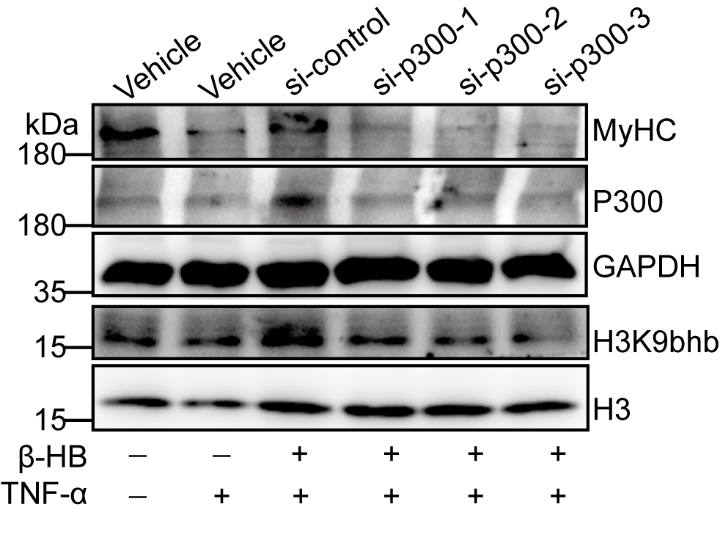
**

**Supplementary Figure 7.** **Knockdown of *p300* by siRNA transfection diminishes the protective effects of β-HB on TNF-α-induced MyHC damage.** C2C12 myotubes were subjected to treatment with TNF-α and β-HB, followed by transfection with si-*p300*. Subsequently, the protein levels of MyHC, p300, and H3K9bhb were assessed through western blot analysis.

**
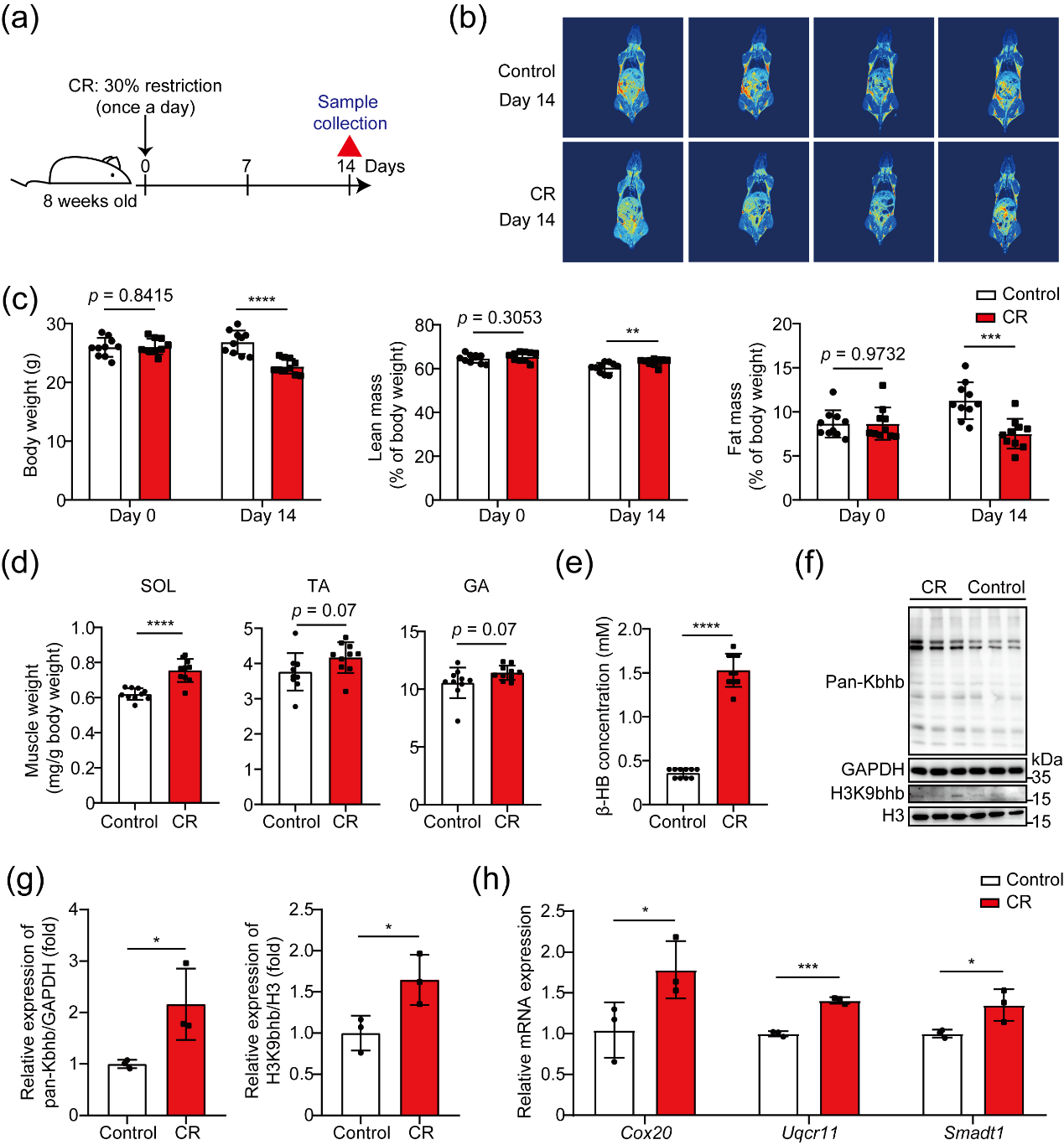
**

**Supplementary Figure 8. CR increases histone Kbhb in the skeletal muscle of mice. (a)** Schematic diagram illustrating the experimental design of 14 days CR in 8-week-old mice. **(b)** Representative images of control and CR mice analyzed by NMR testing. **(c)** Comparison of day 0 and day 14 body weight, relative lean and fat mass for control and CR m​i​c​e. (n = 10) **(d)** Relative weight of SOL GA and TA muscle in control and CR mice. (n = 10). **(e)** Concentration of β-HB in control and CR mice serum. (n = 10). **(f)** Western blot analysis of pan-Kbhb and H3K9bhb in GA muscle of control and CR mice. **(g)** Results in (f) were quantified by Image J. (n = 3). **(h)** The expression levels of mitochondrial pathway-related genes in the GA muscle of control and CR mice were measured by qPCR. (n = 3). Values expressed as the mean ± SD. Significance determined using unpaired *t* test (c, d, e, g and h); **p* < 0.05, ***p* < 0.01, ****p* < 0.001 and *****p* < 0.0001.

**Supplementary Table 1. Comparison of transcription levels of β-HB de novo synthesis pathway in mice and macaques at young and old age, the data are from Aging Atlas.**

| Symbol | Tissue | Species | Treatment | log2 Fold Change | *p* value |
| --- | --- | --- | --- | --- | --- |
| *Acat1*  *Hmgcs2*  *HMGCS2*  *ACAT1* | Liver  Liver  Liver  Liver | *Mus musculus*  *Mus musculus*  *Macaca fascicularis*  *Macaca fascicularis* | Old vs Young  Old vs Young  Old vs Young  Old vs Young | -0.52  -0.63  -0.71  -0.83 | 0.00035  6.03E-05  0.037  0.0001 |
